# Efficacy and Safety of Prothrombin Complex Concentrates in Liver Transplantation: Evidence from Observational Studies

**DOI:** 10.3390/jcm12113749

**Published:** 2023-05-29

**Authors:** Giovanni Punzo, Valeria Di Franco, Valter Perilli, Teresa Sacco, Liliana Sollazzi, Paola Aceto

**Affiliations:** 1 Dipartimento di Scienze dell’Emergenza, Anestesiologiche e della Rianimazione, Fondazione Policlinico Universitario A. Gemelli IRCCS, 00168 Rome, Italy; giovanni.punzo@policlinicogemelli.it (G.P.); valter.perilli@libero.it (V.P.); saccoteresa@gmail.com (T.S.); liliana.sollazzi@unicatt.it (L.S.); 2 Dipartimento di Scienze Biotecnologiche di Base, Cliniche Intensivologiche e Perioperatorie, Università Cattolica del Sacro Cuore, 00168 Rome, Italy

**Keywords:** liver transplantation, prothrombin complex concentrate, blood transfusion, systematic review, meta-analysis

## Abstract

The risk/benefit ratio of using prothrombin complex concentrates (PCCs) to correct coagulation defects in patients with end-stage liver disease is still unclear. The primary aim of this review was to assess the clinical effectiveness of PCCs in reducing transfusion requirements in patients undergoing liver transplantation (LT). This systematic review of non-randomized clinical trials was performed according to the Preferred Reporting Items for Systematic Reviews and Meta-Analyses guidelines. The protocol was previously registered (PROSPERO:CRD42022357627). The primary outcome was the mean number of transfused units for each blood product, including red blood cells (RBCs), fresh frozen plasma, platelets, and cryoprecipitate. Secondary outcomes included the incidence of arterial thrombosis, acute kidney injury, and haemodialysis, and hospital and intensive care unit length of stay. There were 638 patients from 4 studies considered for meta-analysis. PCC use did not affect blood product transfusions. Sensitivity analysis, including only four-factor PCC, showed a significant reduction of RBC effect size (MD: 2.06; 95%CI: 1.27–2.84) with no true heterogeneity. No significant differences in secondary outcomes were detected. Preliminary evidence indicated a lack of PCC efficacy in reducing blood product transfusions during LT, but further investigation is needed. In particular, future studies should be tailored to establish if LT patients will likely benefit from four-factor PCC therapy.

## 1. Introduction

In the last few years, prothrombin complex concentrates (PCC), comprising of a heterogeneous group of plasma-derived products with partly purified vitamin K-dependent clotting factors, have been increasingly used to correct coagulation abnormalities in many clinical settings—including in liver transplantation (LT) [1,2,3,4,5,6,7,8,9]. The key reason for this practice lies in the supposed advantage of reducing blood product transfusions, including fresh frozen plasma (FFP) and red blood cells (RBCs). RBCs are likely to cause transfusion-related acute lung injury (TRALI), possibly due to the presence of antibodies in the donor plasma that react with antigens on the patient’s lung cells [10]. Since PCCs are free of leukocytes and alloantibodies, TRALI has never been associated with intraoperative PCC administration [10].

FFP transfusions, on the other hand, have been associated with several complications, including transfusion-related circulatory overload (TACO) [11,12,13,14,15,16,17,18]. In LT patients, TACO may paradoxically increase surgical bleeding and, consequently, reduce survival by worsening portal hypertension, especially in the pre-anhepatic phase of LTs [2,13,18,19,20,21]. Furthermore, excessive FFP transfusion during the neo-anhepatic phase of LTs can lead to fluid overload, increased central venous pressure, and compromised graft perfusion [2,11,13,18]. As PCCs can be administered in low injection volumes, the use of these drugs in LTs may allow for rapidly raising plasma levels of vitamin K-dependent clotting factors without the risk of volume overload.

Another significant advantage of PCC use in surgical patients, including those undergoing an LT, is a shorter time of administration compared to FFP. Indeed, unlike FFP, PCCs are usually stored at room temperature and do not require thawing. Thus, PCCs can be quickly administered to the patient, allowing a rapid correction of the coagulation defects frequently occurring during LTs. Pharmacological data demonstrate the rapid normalization of vitamin K-dependent clotting factors within 30 min using PCCs in warfarin-treated patients, while FFP transfusion requires at least 3 h to produce the same effect [22,23]. Therefore, PCCs are also supposed to allow a markedly faster and more sustained rise in plasma levels of clotting factors compared to FFP [22,23,24,25].

However, since PCCs are mainly a pro-coagulant therapy, their use has raised concerns about the risk of thrombotic events. Indeed, when LT recipients require significant volume resuscitation, the administration of crystalloid and PCCs should be carefully considered, as it could lead to very low antithrombin levels and increased thrombotic risk. Therefore, in the context of end-stage liver disease (ESLD) coagulopathy, which is characterized by a loss of both pro-coagulant and anticoagulant factors, FFP is often preferred because it can restore coagulation function without increasing the risk of thrombosis [26].

Besides its potential for thromboembolic complications, another major concern for PCC use in LT recipients is [27,28] the risk for acute kidney injury (AKI) and haemodialysis.

Despite the theoretical advantages of PCC use in LT patients, the risk/benefit ratio has never been really explored. The aim of this systematic review and meta-analysis was to assess the clinical effectiveness of PCCs in reducing allogenic blood product transfusions in patients undergoing LT. Our secondary aim was to evaluate safety outcomes, including thrombosis and the incidence of AKI and haemodialysis.

## 2. Materials and Methods

### 2.1. Search Strategy and Studies Selection

This systematic review and meta-analysis were performed following a protocol registered on PROSPERO (ID: CRD42022357627) and reported according to the Preferred Reporting Items for Systematic Reviews and Meta-Analyses (PRISMA) guidelines [29]. The literature search was conducted using computerized databases, including *PubMed*/*MEDLINE*, *The Cochrane Library*, *EMBASE*, and *Scopus*, in order to identify the relevant articles published up to 5 August 2022. Articles were retrieved using the following keywords: “prothrombin complex concentrate” and “liver transplantation”. The full search strategy is available in Document S1 (see Online Supplementary Content). All cohort studies (prospective and retrospective) were considered eligible for inclusion. Inclusion criteria were: human adult subjects and perioperative use of PCCs. Review articles, editorials or letters, comments, and case reports/case series were excluded. Titles and/or abstracts were read thoroughly before complete articles were obtained and the references from the relevant publications were explored to ascertain further potential articles. Abstracts of studies retrieved using the search strategy and those from reference list checks were screened independently by two reviewers (PA, VDF) to identify studies that could potentially meet the inclusion criteria outlined above. The full texts of potentially eligible studies were then retrieved and independently assessed for eligibility by two review team members (PA, VDF). Any disagreement over the eligibility of particular studies was resolved via discussion, also involving a third review author (GP) when necessary.

### 2.2. Data Extraction and Quality Assessment

A standardized table was used to extract data from the included studies. The following data were extracted for each study: first author; year of publication; country; study design; sample size; age; gender (% male); MELD (Model for End-Stage Liver disease); type of donor; PCC product; PCC dose; description of primary and secondary outcomes; mean number of RBC; FFP; PLT and cryoprecipitate units; incidence of thrombosis; AKI and haemodialysis (HD); and hospital and intensive care unit (ICU) length of stay (LOS). Two review authors (PA, VDF) extracted data independently, while discrepancies were identified and resolved through discussion with a third author (GP) when necessary. Original investigators were contacted to request missing data, if necessary. Quality assessment of the included studies was performed using the Newcastle–Ottawa scale [30].

### 2.3. Sensitivity Analyses

Studies on living donors were analysed separately when data on investigated outcomes were available. Sensitivity analyses were also performed, limiting the studies to only those with patients having a mean MELD score > 25 [31] and those that specified the type of PCC product administered.

### 2.4. Data Synthesis and Meta-Analysis

We compared the mean number of units for each blood product transfused during the perioperative period (from the beginning of surgery to the first 48 h in the postoperative period) between the two groups (PCCs versus no PCCs). Primary (mean number of RBCs, FFP, PLTs, and cryoprecipitate units) and secondary outcomes (arterial thrombosis, incidence of AKI and haemodialysis, and hospital and ICU LOS) were analysed as dichotomous or continuous variables, when available. AKI was defined as a change in serum creatinine greater than 0.3 mg/dL within 48 h and a urine output of less than 0.5 mL/kg per hour for 6 to 12 h [32]. For the meta-analyses, we considered the outcome data after matching analyses, when available.

Mean difference (MD) or odds ratio (OR)- with the associated 95% confidence interval (CI)- were calculated, respectively, for continuous and dichotomous outcomes. Data were combined using a random-effects model with the Mantel–Haenszel method. Heterogeneity was described as an I^2^-test. Mean and standard deviation were estimated for studies that only reported median and interquartile range using the estimation method proposed by Wan et al. [33] to allow the pooling of continuous outcomes. Data were analysed using the Review Manager software (Review Manager (RevMan) [Computer program]. Version 5.4. The Cochrane Collaboration, 2020) and STATA 16.1 (StataCorp. 2019. Stata Statistical Software: Release 16. College Station, TX, USA: StataCorp LLC). A *p*-value < 0.05 was considered to be statistically significant.

## 3. Results

### 3.1. Studies Selection

**Figure 1:**
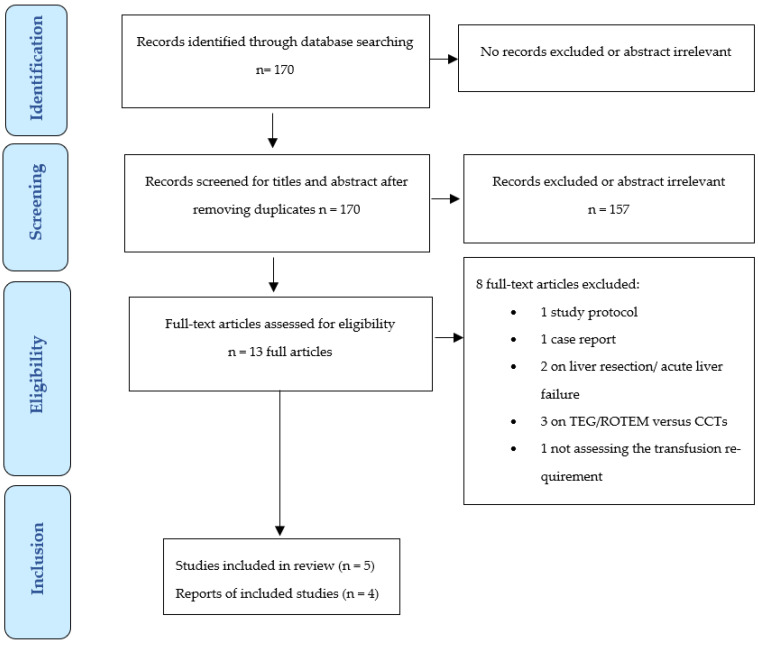
PRISMA flow diagram.

Based on the initial search results, 170 titles and abstracts were examined. No additional publication was retrieved by hand search of the references. 157 reports were rejected because they were not relevant to our study focus. Of the remaining 13 articles, 8 did not meet the inclusion criteria: 1 study protocol, 1 case report, 2 on liver resection/acute liver failure, 3 on viscoelastic versus conventional coagulation tests, and 1 not assessing transfusion requirement. In the end, five publications were reviewed, four of which were included in the meta-analysis (see Figure 1). One study was excluded from the quantitative analysis because PCC was only administered in a sub-population of LT recipients to reverse anti-vitamin k agents prior to LT [34].

### 3.2. Study Characteristics

The main characteristics of the studies are shown in Table 1. All included studies were observational, four were retrospective and one was a before–after study. The studies (n = 4) used for the quantitative analysis involved a total of 638 participants, including 301 patients with PCC exposure and 337 who did not receive PCCs. The total number of female patients was 211, equal to 33.1% of the sample (M/F ratio 211/427). The mean age was 52.5 (standard deviation, SD: 9.87) years and the mean MELD score was 25.89 (SD: 9.83). Four-factor PCC (Beriplex^®^/Kcentra^®^, CSL Behring GmbH, Marburg, Germany) was administered in three studies [4,5,34], while two studies [6,7] did not report the type of PCC used. In all studies, viscoelastic tests guided perioperative coagulation management. In addition, standard clinical assessments and conventional coagulation tests were performed on all patients.

### 3.3. Primary Outcomes

Study outcomes are presented in Table 2.

**Figure 2:**
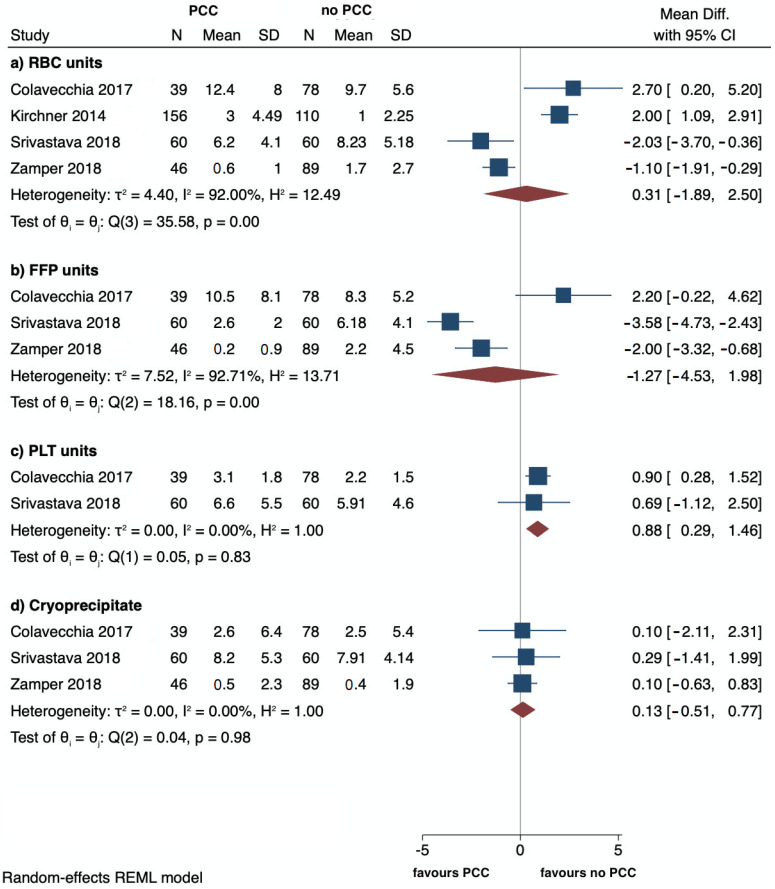
Forest plots reporting transfused units of RBCs, FFP, PLTs, and cryoprecipitate, respectively, (**a**–**d**) in PCC versus no PCC groups. The effect size is calculated as a mean difference (MD) and corresponding 95% confidence interval (95%CI) [4,5,6,7].

The mean number of PLT units transfused was greater (MD: 0.88, 95%CI: 0.29 to 1.46; I^2^ = 0%) in the PCC group than in the group not receiving PCCs (Figure 2c). There was no difference for RBC (MD: 0.31, 95%: −1.89 to 2.50; I^2^ = 92%), FFP (MD: −1.27, 95%: −4.53 to 1.98; I^2^ = 92.7%), and cryoprecipitate (MD:0.13, 95%: −0.51 to 0.77; I^2^ = 0%) units transfused between the two groups (Figure 2a,b,d).

### 3.4. Secondary Outcomes

**Figure 3:**
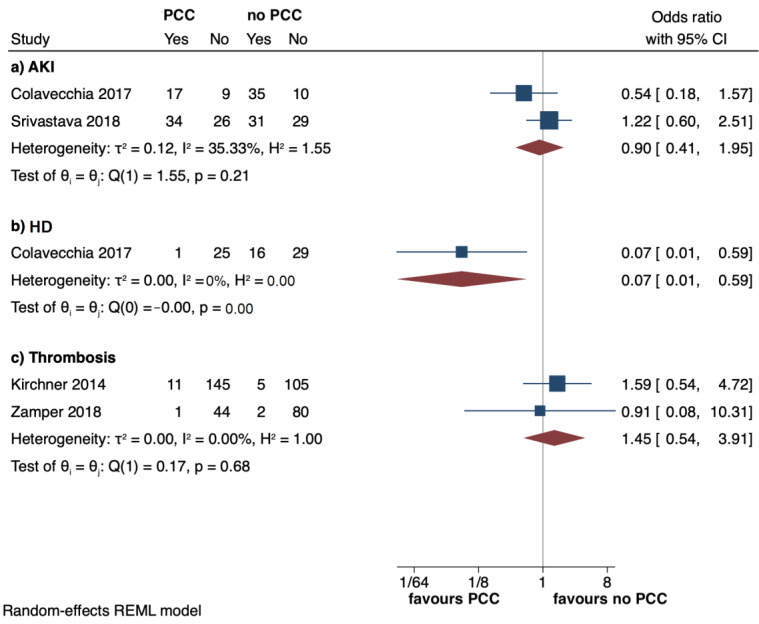
Forest plots reporting acute kidney injury (AKI), haemodialysis (HD), arterial thrombosis, respectively, (**a**–**c**) in PCC versus no PCC groups. The effect size is calculated as an odds ratio (OR) and corresponding 95% confidence interval (95%CI) [4,5,6,7].

**Figure 4:**
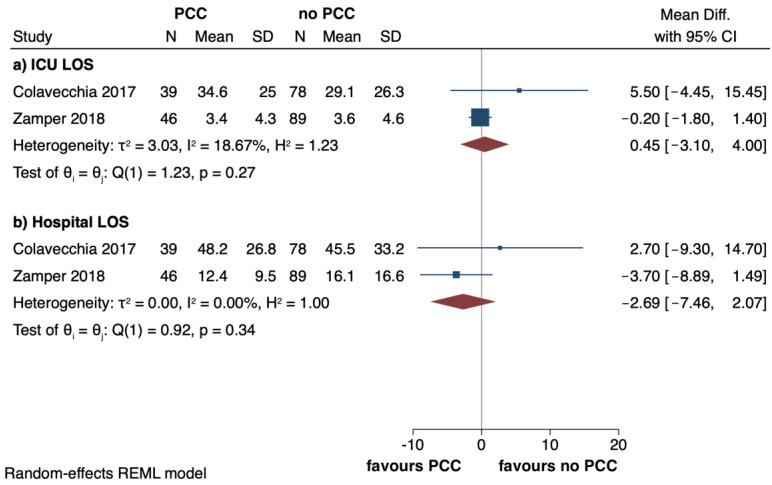
Forest plots reporting intensive care unit (ICU) and hospital length of stay (LOS), respectively, (**a**,**b**) in PCC versus no PCC groups. The effect size is calculated as a mean difference (MD) and corresponding 95% confidence interval (95%CI) [5,7].

Analyses showed a non-significant effect size for the incidence of AKI (OR: 0.90, 95%: 0.41 to 1.95; I^2^ = 35%) and arterial thrombosis (OR: 1.45, 95%: 0.54 to 3.91; I^2^ = 0%) between the PCC exposure cohort and the one without PCCs (Figure 3a,c). Only one study [5] showed a reduced incidence of haemodialysis in the PCC group (3.8% vs 35.6%) (Figure 3b). ICU (MD: 0.45, 95%: −3.10 to 4; I^2^ = 18.7%) and hospital (MD: −2.69, 95%: −7.46 to 2.07; I^2^ = 0%) LOS’ were similar in the two groups (Figure 4a,b).

### 3.5. Sensitivity Analyses

Sensitivity analyses, excluding a study on living donors, [6] confirmed the absence of statistical significance with similar heterogeneity for RBC (*p* = 0.43, I^2^ = 95%), FFP (*p* = 0.96, I^2^ =87%), and cryoprecipitate units (*p* = 0.79, I^2^ = 0%) (see Figures S1–S3). Sensitivity analyses, including studies reporting a MELD score > 25 [5,6], did not show significant results for RBC (MD: 0.20; 95%CI, −4.43 to 4.82; *p* = 0.93; I^2^ = 88%), FFP (MD: −0.84; 95%CI, −6.49 to 4.82; *p* = 0.77; I^2^ = 93%), and cryoprecipitate (MD: 0.22; 95%CI, −1.15 to 1.60; *p* = 0.75; I^2^ = 0%) units, respectively (see Figures S4–S6). Sensitivity analyses, including studies reporting the type of PCC product administered (four-factor PCC), showed a significant effect size (MD: 2.06; 95%CI, 1.27 to 2.84; *p* < 0.00001) with no true heterogeneity (I^2^ = 0%) for fewer RBC units transfused in the PCC group (see Figure S7). For the other outcomes, an insufficient number of studies was available to perform any sensitivity analysis.

### 3.6. Risk of Bias

Quality ratings of the studies averaged 84% (range = 0.6–1) of the maximum attainable score with the Newcastle—Ottawa Scale (see Table S1). Matching analysis was used in three studies [5,6,7] to reduce selection bias.

## 4. Discussion

The results of this systematic review and meta-analysis, including 638 participants from 4 observational studies, found that the use of PCCs did not decrease the transfusions of allogenic blood products, including RBCs, FFP, and cryoprecipitate. However, a sensitivity analysis on two studies suggested that four-factor PCC was effective in reducing RBC transfusion, making us suppose that the use of this product could be further explored. The non-negligible risk of bias in the available studies and the paucity of data on some of the selected outcomes significantly lowered the strength of the study results (particularly regarding FFP and cryoprecipitate transfusions). Additionally, the increased significant PLT consumption observed in the PCC group lacked sufficient evidence to draw firm conclusions.

In patients undergoing LT, the transfusion of allogenic blood products (particularly of RBCs) has been associated with an increased morbidity and mortality [1,2,4,35,36,37,38]. During LT, intraoperative bleeding, which consequently requires RBC transfusion, relies on patient-related factors, surgical factors, and coagulation management strategies. Indeed, in LT patients, the transfusion of large volumes of FFP for the correction of a severe coagulopathy may lead to fluid overload, increased venous pressure, portal hypertension, and increased bleeding from the surgical field [2,4,5,6,11,12,13]. Hence, a “volume-restrictive substitution therapy” was suggested for patients undergoing LT, mainly for the dissection phase of surgery [1,2,11,12,13,37].

Theoretically, due to their pharmacological properties, PCCs could be ideal to prevent excessive fluid administration in patients with severe coagulopathy in surgical settings. Indeed, the recommended dose of FFP to correct INR to the target value of 1.5 is around 10–30 mL/kg for most patients with severe coagulopathy and increased surgical bleeding [1,2,11,19]. As the administration of 1 UI/kg of PCC usually increases the activity of corresponding coagulation factors by 0.6–1%, the same effect of the above-mentioned large volume of plasma can be accomplished with a dose of PCC not exceeding 10–30 UI/kg. This dose can be delivered in injection volumes of no more than 1–2 ml/kg, which are generally well-tolerated by surgical patients [12].

In a recent meta-analysis of a total of 3060 patients with major bleeding, PCC administration reduced the need for RBC transfusions when compared with treatment strategies not involving PCCs [36]. Moreover, PCC administration—when added to FFP—was associated with reduced mortality in the subgroup of trauma patients without increasing thromboembolic events [36].

Large studies on PCC use in patients undergoing LT are rare, and there is no published randomized controlled trial on the topic. Indeed, the results of the ongoing PROTON trial, which is the only such trial in Europe, are not available yet [35]. Therefore, the efficacy and safety of PCCs in patients undergoing LT remain unclear, and this is the first systematic review and meta-analysis which addresses these issues.

In our meta-analysis, PCC use does not reduce perioperative RBC and FFP transfusions in the perioperative period of LT. This result is influenced by two studies [4,5], which require, in our opinion, specific considerations.

Indeed, our analysis revealed that, in the study of Colavecchia and colleagues [5], the use of PCCs was not regulated by any standardized protocol, and providers could freely decide on type, dose, time of administration of these drugs, concomitant use of allogenic blood products, and coagulation monitoring modalities (viscoelastic vs conventional coagulation tests) exclusively according to their clinical judgment. Due to the severity of the hepatopathy in the study population (mean MELD scores in the cohort of patients treated with PCCs was 34.4; SD: 9.2), almost certainly Colavecchia and colleagues had to manage patients with a near-global coagulation factor deficiency. In this case, supplementation of vitamin K-dependent factors alone (by PCC administration) could be unable to restore a pro-coagulant state [5]. Their patients were likely unresponsive to the first PCC dose, and this may have led the authors, in the absence of a strictly defined protocol, to frequently abandon the experimental treatment with PCCs [5]. In fact, PCCs were often given as a single dose (median PCC dose was 32.1 UI/kg) at the beginning of the procedure, typically within 1.5 h from skin incision [5]. Conversely, a large number of FFP units were transfused in the PCC group (10.5 ± 8.1) [5].

Regarding the study of Kirchner and colleagues [4], it must be noted that the non-PCC group included almost exclusively patients who generally did not receive any haemostatic therapy. The main aim of this study was to evaluate the risk for thromboembolic complications in patients treated with PCCs, and only patients without widespread bleeding—and with very low hemostatic requirements—were retrospectively included by the authors in the non-PCC group [4]. The median MELD score in the non-PCC group was 17 (vs. 23 in the PCC group), and only 6.4% of patients in this group received FFP transfusions [4]. The mean number of FFP and RBC units transfused in the non-PCC group was so low (close to 0), that it could not be lowered [4]. This could explain why the authors did not find any reduction, but an increase in the mean number of FFP and RBC units transfused in the PCC group compared to the non-PCC group [4].

Based on these considerations, it is possible that these two studies [4,5] underestimated the actual effects of PCCs on allogenic blood product consumption in patients undergoing LT. Therefore, this meta-analysis could result in an under-representation of the effectiveness of PCC use in reducing allogenic blood transfusions in LTs.

In our meta-analysis, PLT consumption was significantly greater in patients treated with PCCs than in patients not treated with these drugs. However, this result must be interpreted with great caution, because only two eligible studies reported data on PLT consumption in LT patients receiving PCCs [5,6].

Of note, the results of our meta-analysis on PLT consumption are strictly influenced by the study of Colavecchia and colleagues [5], in which the severity of liver disease in the study population was very high (mean MELD score in the PCC group was 34.4). Their patients likely had a severe deficit of almost all coagulation factors, including Factors V, XI, and XIII. As platelet α-granules are known to contain Factor V (which is not present in PCC formulations), this may explain the greater PLT consumption in these patients [5].

Finally, in regards to safety, the administration of PCCs did not result in an increase of thromboembolic complications, and AKI incidence was similar in patients treated or not with these drugs. Even if the CIs of the ORs were very wide, suggesting differential results across studies, these findings are in line with that of a recent meta-analysis on PCC use in patients with severe bleeding [36].

From a clinical perspective, both arterial and portal vein thrombosis are devastating complications of LT, feared much more than TACO and TRALI. This may have led many anesthesiologists and transplant surgeons, especially in the pre- four-factor PCC era, to frequently tolerate a mild coagulopathy compared to the overcorrection of haemostasis. Hence, data on PCC use in the setting of LTs are scarce, and evidence that these drugs are not associated with thrombotic or thromboembolic events is currently low [39].

In LT patients, viscoelastic tests (VETs)-guided coagulation management seems to be associated with an increased PCC administration compared to standard coagulation tests [39], but the incidence of thrombotic complications in the VET group was evaluated in most studies as secondary or exploratory outcomes, with no adjustments made for multiple comparisons.

This study has several limitations. First, our analysis is limited by the current absence of RCTs comparing PCC-based with standard FFP-based algorithms for coagulation management in patients undergoing LT. All studies included in this meta-analysis were observational and monocentric, with a retrospective or before-and-after design. This certainly contributed to an increased risk of bias. However, the inclusion of three propensity-matched studies in this meta-analysis provides, up to now, the best available evidence on PCC use during LT [5,6,7]. Second, coagulation management algorithms were heterogeneous among eligible studies in both the PCC and non-PCC groups. There is a considerable variety between studies in the type and dose of PCCs used, as well as in the trigger and timing for PCC administration. Moreover, the methods used to assess coagulation and guide PCC administration in patients undergoing LT (standard laboratory vs viscoelastic tests) were different among studies, and PCCs were sometimes administered based solely on clinical judgment. This could lead to both an under- or over-representation of the actual effects of the available PCCs, due to inappropriate use or dosing and non-optimal timing of administration of these drugs in patients without a substantial coagulopathy. Researchers should also take into account that the type of test for coagulation monitoring may have a role in affecting transfusion requirements [40], and the type of tool used should be standardized across future studies. Third, not all studies had strict inclusion or exclusion criteria for their sample, leading to a heterogeneous study population. Of note, none of the available studies included screening for underlying patient conditions and safety of PCCs in patients with a hypercoagulable condition—such as hepatocellular carcinoma, Budd-Chiari syndrome, portal vein thrombosis, or primary sclerosing cholangitis—remains unclear. Finally, no differences were detected between patients treated or not with PCCs for overall hospital LOS, even if this finding suffers from the paucity of data extracted only from two studies. Unfortunately, none of the selected studies reported the rate of patient survival, which remains the most important outcome in this group of patients [41,42]. Moreover, data regarding graft failure and postoperative respiratory complications were insufficient to allow an analysis. However, the effect of four-factor PCC on the reduction of transfusion burden, if confirmed by future studies, may lead to positive consequences on recipient survival.

## 5. Conclusions

Based on the current evidence, PCC administration in patients undergoing LT seems not to be associated with reduced RBC and FFP consumption when compared to a resuscitation strategy involving solely FFP, even if four-factor PCC seems to show promising results. Moreover, PCC use in patients undergoing LT seems not to be burdened by thrombotic, thromboembolic, or renal complications. These results must be interpreted with great caution, since they are subject to considerable heterogeneity. Moreover, all available data are derived from small observational studies. The current lack of robust evidence does not allow us to draw firm conclusions on the use of PCCs in patients undergoing LT.

## Figures and Tables

**Table 1:** Characteristics of included studies.

First Author, Year (Country)	Study Design	Sample, n	Age, Mean (SD) or Median [IQR]	Male, n (%)	MELD, Mean (SD) or Median [IQR]	Type of Donor	PCC Product	PCC (UI) and FibC (g) Dose Mean (SD)	Primary Outcome	Secondary Outcome
Colavecchia, 2017 (USA) [5]	R	PCC 39 No PCC 78	55 (11.4) 55.7 (9.44)	23 (58.9) 45 (57.7)	34.4 (9.2) 35.4 (8.8)	D	Kcentra	PCC 902 (589) FibC 1.7 (1.1)	Requirement of RBC	Requirement of FFP, PLT, cryoprecipitate, OR take-backs within 72 h; total LOS; ICU LOS; incidence of AKI and HD
Kirchner, 2014 (Germany) [4]	R	CFCs 156 No CFCs 110	51.2 (10.4) 53 (9.4)	100 (64) 64 (58)	23 [16–33] 17 [11.5–25]	D	Beriplex P/N	PCC 4090 (3130) FibC 6.3 (5.6)	Safety events	Transfusion requirement
Martinez, 2021 (Spain) [34]	R	PCC 11 No PCC 14	59 [57–62] 57 [55–61]	10 (91%) 11 (79%)	19 [17–20] 20 [18–24]	D	Beriplex	Median dose of PCC 600 [IQR 500–1100] IU	Bleeding, transfusion requirements	Safety of PCC administration
Srivastava, 2018 (India) [6]	R	PCC 60 No PCC 60	49.5 (5.7) 51.7 (8.98)	43 (71.7) 46 (76.4)	33.9 (11.4) 32.7 (12.5)	L	NS	Dose of PCC 25 IU/kg	Requirement of RBC	PO days of VM, incidence of AKI, PO hemorrhagic complication
Zamper, 2018 (Brazil) [7]	BAS	PCC 46 No PCC 89	53 (11.8) 52.5 (11.9)	35 (76.1) 71 (79.8)	21.9 (9.2) 22 (7.9)	D	NS	PCC 195.6 (645.3) FibC 1.4 (2.4)	Transfusion requirement	Use of CFCs or antifibrinolytic; clinical complications related to the procedure; PO duration of VM; ICU and hospital LOS; in-hospital mortality

Notes: AKI, acute kidney injury; AVK, Antivitamin K agent; BAS, before–after study; CFCs, coagulation factor concentrates; D, deceased; FFP, fresh frozen plasma; FibC, fibrinogen concentrate; h, hours; HD, hemodialysis; IQR, interquartile range; ICU, intensive care unit; L, living; LOS, length of stay; n, number; NS, not stated; OR, operating room; PLT, platelets; PO, postoperative; PCC, prothrombin complex concentrate; R, retrospective; RBC, red blood cell; SD, standard deviation; IU, international units; g, grams; D, deceased; L, living; MV, mechanical ventilation.

**Table 2:** Study outcomes.

First Author, Year	RBC Mean (SD) or Median [IQR], n (%)	FFP Mean (SD) or Median [IQR], n (%)	PLT Mean (SD), n (%)	FibC or Cryo Mean (SD), n (%)	Safety Events n (%)	AKI n (%)	HD n (%)	Hospital and ICU LOS Mean (SD), n (%)	Others
Colavecchia, 2017 [5]	PCC group 12.4 (8) No PCC group 9.7 (5.6)	PCC group 10.5 (8.1) No PCC group 8.3 (5.2)	PCC group 3.1 (1.8) No PCC group 2.2 (1.5)	Cryo: PCC group 2.6 (6.4) No PCC group 2.5 (5.4)	NS	PCC group 17/26 (65.4) No PCC group 35/45 (77.8)	PCC group 1/26 (3.8) No PCC group 16/45 (35.6)	Hospital LOS: PCC group 48.2 (26.8) No PCC group 45.5 (33.2) ICU LOS: PCC group 34.6 (25) No PCC group 29.1 (26.3)	Operative take-back: PCC group 18 (46.2%) No PCC group 40 (51.3%)
Kirchner, 2014 [4]	CFC group 3 [0–6] 111 (71.2%) No CFC group 0 [0–3] 58 (52.7%)	CFC group 0 [0–0] 32 (20.5%) No CFC group 0 [0–0] 7 (6.4%)	CFC group 0 [0–1] 64 (41%) No CFC group 0 [0–0] 12 (10.9%)	NS	HAT: CFC group 7 (4.5%) No CFC group 4 (3.6%) PVT and MI: CFC group 1 (0.6%) PE: CFC group 2 (1.28%) No CFC group 1 (0.9%) Stroke: no patient Composite outcome: CFC group 11 (7.1%) No CFC group 5 (4.5%)	NS	NS	NS	NS
Martinez, 2021 [34]	PCC group 3.5 [2.2–5.5] 4 (36%) No PCC group 3 [2–7] 7 (50%)	PCC group 500 [500–500] ml 2 (18%) No PCC group 1000 [895–2375] 6 (43%)	PCC group 6 (9%) No PCC group 2 (14%)	FibrC/ cryo: PCC group 6 (54%) No PCC group 8 (57%)	Thrombosis: PCC group 0 No PCC group 1 (7%)	KDIGO 2: PCC group 1 (9) No PCC group 2 (14)	NS	Hospital stay: PCC group 17 (11–31) No PCC group 15 (13–23)	Deaths: PCC group 0 No PCC group 1 (7%) Bleeding: PCC group 2 (18%) No PCC group 1 (7%) Reintervention for bleeding: PCC group 2 (18%) No PCC group 1 (7%)
Srivastava, 2018 [6]	PCC group 6.2 (4.1) No PCC group 8.23 (5.18)	PCC group 2.6 (2) No PCC group 6.18 (4.1)	PCC group 6.6 (5.5) No PCC group 5.91 (4.6)	Cryo: PCC group 8.2 (5.3) No PCC group 7.9 (4.1)	Thrombotic events: PCC group 0 No PCC group 0	PCC group 34 (56.6) No PCC group 31 (51.6)	PCC group 0 No PCC group 0	LOS > 21 days: PCC group 2 (3.3%) No PCC group 3 (5%)	PO MV > 1 day: PCC group 7 (11.6%) No PCC group 8 (13.3%) Hemorrhagic complications: PCC group 2 (3.3%) No PCC group 7 (11.6%)
Zamper, 2018 [7]	PCC group 0.6 (1) No PCC group 1.7 (2.7)	PCC group 0.2 (0.9) No PCC group 2.2 (4.5)	PCC group 0 (0) No PCC group 0.1 (0.6)	Cryo: PCC group 0.5 (2.3) No PCC group 0.4 (1.9) FibC: PCC group 1.4 (2.4) No PCC group 0 (0)	HAT: PCC group 1/45 (2.2) No PCC group 2/82 (2.4)	NS	NS	ICU LOS: PCC group 3.4 (4.3) No PCC group 3.6 (4.6) Hospital LOS: PCC group 12.4 (9.5) No PCC group 16.1 (16.6)	Duration of MV: PCC group 0.5 (1.2) No PCC group 0.9 (1.4) In-hospital mortality: PCC group 1/45 (2.2) No PCC group 5/89 (5.6)

Notes: AKI, acute kidney injury; CFCs, coagulation factor concentrates; FFP, fresh frozen plasma; FibC, Fibrinogen concentrates; Cryo, cryoprecipitate; HAT, hepatic artery thrombosis; HD, hemodialysis; ICU, intensive care unit; LOS, length of stay; n, number; NS, not stated; PE, pulmonary embolism; PLT, platelets; PVT, portal vein thrombosis; RBC, red blood cell; SD, standard deviation; MI, myocardial ischemia; MV, mechanical ventilation; *, it includes thrombotic, thrombo-embolic, and ischemic events.

## Data Availability

Not applicable.

## Supplementary Materials

The following supporting information can be downloaded at: https://www.mdpi.com/article/10.3390/jcm12113749/s1, Document S1: Search Strategy; Figure S1: Forest plot of RBC units excluding study with living donor; Figure S2: Forest plot of FFP units excluding study with living donor; Figure S3: Forest plot of cryoprecipitate units excluding study with living donor; Figure S4: Forest plot of RBC units including studies with MELD score > 25; Figure S5: Forest plot of FFP units including studies with MELD score > 25; Figure S6: Forest plot of cryoprecipitate units including studies with MELD score > 25; Figure S7: Forest plot of RBC units including studies reporting type of PCC product administered; Table S1: Risk of bias table for assessing the quality of cohort studies by using the Newcastle-Ottawa Scale.
