# Supplementary material for: Efficacy and Safety of Prothrombin Complex Concentrates in Liver Transplantation: Evidence from Observational Studies
Source: J Clin Med. 2023 May 29;12(11):3749. doi: 10.3390/jcm12113749 (PMC10253292; doi:10.3390/jcm12113749)
Supplement: Supplementary file 1 [file jcm-12-03749-s001.zip › jcm-2349491-supplementary.pdf]

Online Supplementary Content

**Document S1**	Search Strategy
**Figure S1**	Forest plot of RBC units excluding study with living donor
**Figure S2**	Forest plot of FFP units excluding study with living donor
**Figure S3**	Forest plot of cryoprecipitate units excluding study with living donor
**Figure S4**	Forest plot of RBC units including studies with MELD score > 25
**Figure S5**	Forest plot of FFP units including studies with MELD score > 25
**Figure S6**	Forest plot of cryoprecipitate units including studies with MELD score > 25
**Figure S7**	Forest plot of RBC units including studies reporting type of PCC product administered
**Table S1**	Risk of bias table for assessing the quality of cohort studies by using the Newcastle-Ottawa Scale

Document S1

Search Strategy PCC in liver transplantation

1. **Cochrane CENTRAL search strategy**
   CENTRAL (via onlinelibrary.wiley.com)

#1 ("prothrombin complex concentrate"):ti,ab,kw
#2 (pcc*):ti,ab,kw
#3 #1 OR #2
#4 liver OR hepatic OR hepato
#5 MeSH descriptor: [Liver] explode all trees
#6 #4 OR #5
#7 resection OR segmentectomy OR graft* OR transplant*
#8 #6 AND #7
#9 hepatectomy
#10 MeSH descriptor: [Hepatectomy] explode all trees
#11 MeSH descriptor: [Liver Transplantation] explode all trees
#12 #8 OR #9 OR #10 OR #11
#13 #3 AND #12

2. **Pubmed/MEDLINE search strategy**
   MEDLINE 1946 to 5th August 2022.

(prothrombin complex concentrate) AND (((liver OR hepatic OR hepato OR “liver”[MeSH]) AND (resection OR segmentectomy OR graft* OR transplant*)) OR hepatectomy OR “hepatectomy”[MeSH] OR “Liver Transplantation”[MeSH])

3. **EMBASE search strategy**
   EMBASE (OvidSP)
   Database: Embase Classic + Embase, 1947 to 5th August 2022.

#1 prothrombin complex concentrate.tw.
#2 pcc*.tw.
#3 #1 or #2
#4 (liver or hepatic or hepato).af.
#5 (segmentectomy or resection or graft* or transplant*).af.
#6 #4 and #5
#7 hepatectomy.af.
#8 exp liver resection/ or exp liver transplantation/
#9 #6 or #7 or #8
#10 #3 AND #9

4. **Scopus Database, search strategy, up to 5th August 2022**

"prothrombin complex concentrate" AND "Liver Transplant"

Figure S1. Forest plot of RBC units excluding study with living donor

The results show a non-significant effect size favoring the use of PCC. The effect size is reported as mean difference with the corresponding 95% confidence interval (95% CI).

	PCC			No PCC				Mean Difference
Study or Subgroup	Mean	SD	Total	Mean	SD	Total	Weight	IV, Random, 95% CI
Colavecchia 2017	12.4	8	39	9.7	5.6	78	26.2%	2.70 [-0.10, 5.50]
Kirchner 2014	3	4.49	156	1	2.25	110	36.6%	2.00 [1.18, 2.82]
Zamper 2018	0.6	1	46	1.7	2.7	89	37.2%	-1.10 [-1.73, -0.47]
**Total (95% CI)**			**241**			**277**	**100.0%**	**1.03 [-1.54, 3.60]**

Heterogeneity: $Tau^2 = 4.53$; $Chi^2 = 37.78$, df = 2 ($P < 0.00001$); $I^2 = 95\%$
Test for overall effect: Z = 0.79 (P = 0.43)

Mean Difference
IV, Random, 95% CI

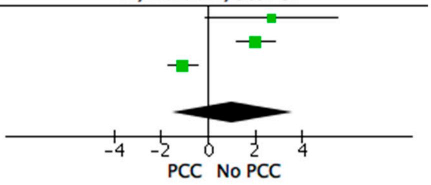

Figure S2. Forest plot of FFP units excluding study with living donor

The results show a non-significant effect size favoring the use of PCC. The effect size is reported as mean difference with the corresponding 95% confidence interval (95% CI).

	PCC			No PCC				Mean Difference
Study or Subgroup	Mean	SD	Total	Mean	SD	Total	Weight	IV, Random, 95% CI
Colavecchia 2017	10.5	8.1	39	8.3	5.2	78	44.9%	2.20 [-0.59, 4.99]
Zamper 2018	0.2	0.9	46	2.2	4.5	89	55.1%	-2.00 [-2.97, -1.03]
**Total (95% CI)**			**85**			**167**	**100.0%**	**-0.11 [-4.21, 3.98]**

Heterogeneity: $Tau^2 = 7.68$; $Chi^2 = 7.76$, df = 1 ($P = 0.005$); $I^2 = 87\%$
Test for overall effect: Z = 0.05 (P = 0.96)

Mean Difference
IV, Random, 95% CI

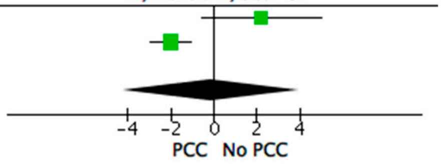

Figure S3. Forest plot of cryoprecipitate units excluding study with living donor

The results show a non-significant effect size favoring the use of PCC. The effect size is reported as mean difference with the corresponding 95% confidence interval (95% CI).

	PCC			No PCC				Mean Difference
Study or Subgroup	Mean	SD	Total	Mean	SD	Total	Weight	IV, Random, 95% CI
Colavecchia 2017	2.6	6.4	39	2.5	5.4	78	9.8%	0.10 [-2.24, 2.44]
Zamper 2018	0.5	2.3	46	0.4	1.9	89	90.2%	0.10 [-0.67, 0.87]
**Total (95% CI)**			**85**			**167**	**100.0%**	**0.10 [-0.63, 0.83]**

Heterogeneity: $Tau^2 = 0.00$; $Chi^2 = 0.00$, df = 1 ($P = 1.00$); $I^2 = 0\%$
Test for overall effect: Z = 0.27 (P = 0.79)

Mean Difference
IV, Random, 95% CI

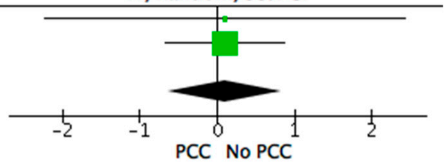

Figure S4. Forest plot of RBC units including studies with MELD score > 25

The results show a non-significant effect size favoring the use of PCC. The effect size is reported as mean difference with the corresponding 95% confidence interval (95% CI).

Study or Subgroup	PCC Mean	PCC SD	PCC Total	No PCC Mean	No PCC SD	No PCC Total	Weight	Mean Difference IV, Random, 95% CI
Colavecchia 2017	12.4	8	39	9.7	5.6	78	47.1%	2.70 [-0.10, 5.50]
Srivastava 2018	6.2	4.1	60	8.23	5.18	60	52.9%	-2.03 [-3.70, -0.36]
**Total (95% CI)**			**99**			**138**	**100.0%**	**0.20 [-4.43, 4.82]**

Heterogeneity: Tau² = 9.80; Chi² = 8.08, df = 1 (P = 0.004); I² = 88%
Test for overall effect: Z = 0.08 (P = 0.93)

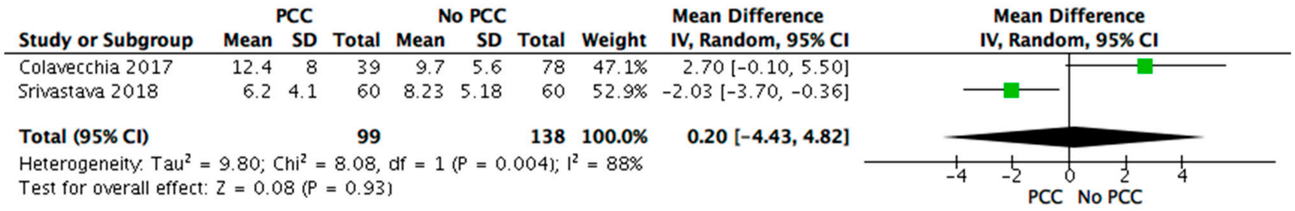

Figure S5. Forest plot of FFP units including studies with MELD score > 25

The results show a non-significant effect size favoring the use of PCC. The effect size is reported as mean difference with the corresponding 95% confidence interval (95% CI).

Study or Subgroup	PCC Mean	PCC SD	PCC Total	No PCC Mean	No PCC SD	No PCC Total	Weight	Mean Difference IV, Random, 95% CI
Colavecchia 2017	10.5	8.1	39	8.3	5.2	78	47.5%	2.20 [-0.59, 4.99]
Srivastava 2018	2.6	2	60	6.18	4.1	60	52.5%	-3.58 [-4.73, -2.43]
**Total (95% CI)**			**99**			**138**	**100.0%**	**-0.84 [-6.49, 4.82]**

Heterogeneity: Tau² = 15.52; Chi² = 14.06, df = 1 (P = 0.0002); I² = 93%
Test for overall effect: Z = 0.29 (P = 0.77)

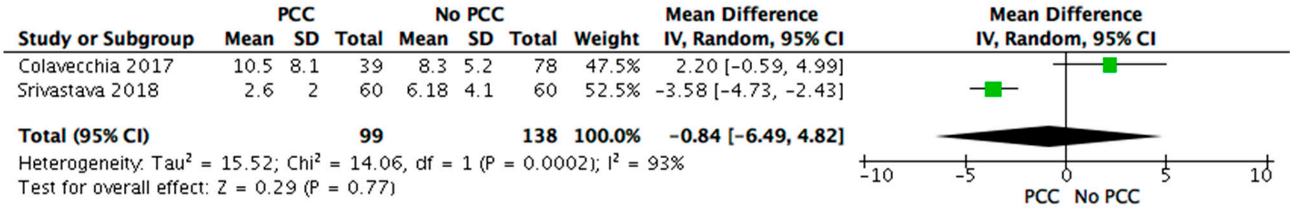

Figure S6. Forest plot of cryoprecipitate units including studies with MELD score > 25

The results show a non-significant effect size favoring the use of PCC. The effect size is reported as mean difference with the corresponding 95% confidence interval (95% CI).

Study or Subgroup	PCC Mean	PCC SD	PCC Total	No PCC Mean	No PCC SD	No PCC Total	Weight	Mean Difference IV, Random, 95% CI
Colavecchia 2017	2.6	6.4	39	2.5	5.4	78	34.6%	0.10 [-2.24, 2.44]
Srivastava 2018	8.2	5.3	60	7.91	4.14	60	65.4%	0.29 [-1.41, 1.99]
**Total (95% CI)**			**99**			**138**	**100.0%**	**0.22 [-1.15, 1.60]**

Heterogeneity: Tau² = 0.00; Chi² = 0.02, df = 1 (P = 0.90); I² = 0%
Test for overall effect: Z = 0.32 (P = 0.75)

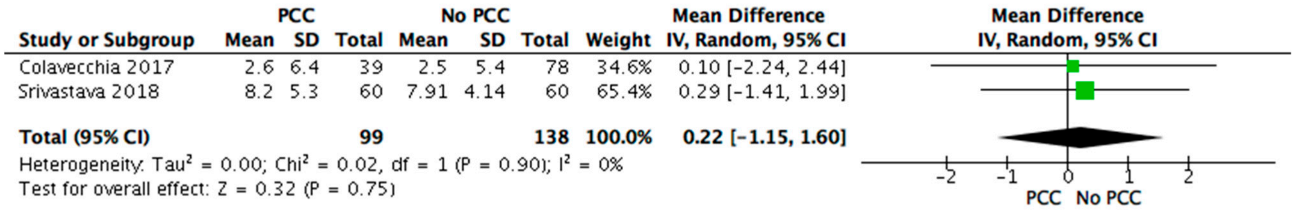

Figure S7. Forest plot of RBC units including studies reporting type of PCC product administered

The results show a significant effect size favoring the use of PCC. The effect size is reported as mean difference with the corresponding 95% confidence interval (95% CI).

Study or Subgroup	PCC Mean	PCC SD	PCC Total	No PCC Mean	No PCC SD	No PCC Total	Weight	Mean Difference IV, Random, 95% CI
Colavecchia 2017	12.4	8	39	9.7	5.6	78	7.9%	2.70 [-0.10, 5.50]
Kirchner 2014	3	4.49	156	1	2.25	110	92.1%	2.00 [1.18, 2.82]
**Total (95% CI)**			**195**			**188**	**100.0%**	**2.06 [1.27, 2.84]**

Heterogeneity: Tau² = 0.00; Chi² = 0.22, df = 1 (P = 0.64); I² = 0%
Test for overall effect: Z = 5.12 (P < 0.00001)

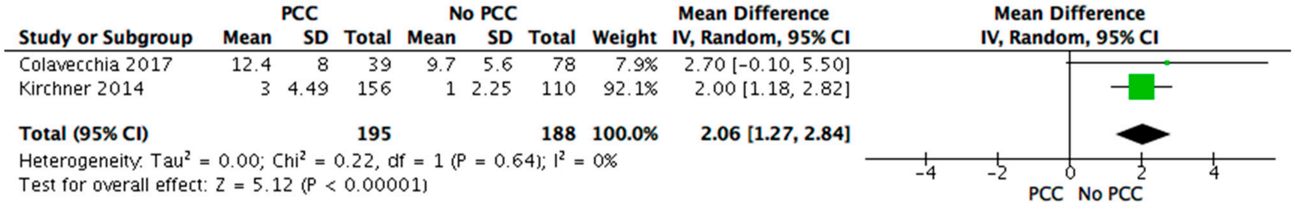

Table S1. Risk of bias table for assessing the quality of cohort studies by using the Newcastle-Ottawa Scale

	Representativenes of the exposed cohort	Selection of the non exposed cohort	Ascertainment of exposure	Demonstration that outcome of interest was not present at start of study	Comparability of cohorts on the basis of the gender	Comparability of cohorts on the basis of the age	Comparability of cohorts on the basis of the MELD score	Assessment of outcome	Was follow-up long enough for outcomes to occur	Adequacy of follow up of cohorts
**Colavecchia, 2017**	*	*	*	*	*	*	*	*		*
**Kirchner, 2014**			*	*	*	*		*		*
**Martinez, 2021**			*	*	*	*	*	*	*	*
**Srivastava, 2018**	*	*	*	*	*	*	*	*	*	*
**Zamper, 2018**	*	*	*	*	*	*	*	*		*
